# Supplementary material for: Effectiveness of a peer educator-coordinated preference-based differentiated service delivery model on viral suppression among young people living with HIV in Lesotho: The PEBRA cluster-randomized trial
Source: PLoS Med. 2023 Jan 3;20(1):e1004150. doi: 10.1371/journal.pmed.1004150 (PMC9810159; doi:10.1371/journal.pmed.1004150)
Supplement: S1 Table — (DOCX) [file pmed.1004150.s002.docx]

**Table S1.** Details about each PEBRA intervention component option

| **Option** | **Explanation** | **Frequency and timing** | **SMS notification content** |
| --- | --- | --- | --- |
| **Component 1: ART refill** | | | |
| At the clinic | The participant picks up ART at the clinic. This includes pick-up at the clinic-based Saturday Clinic Club. | 3-6 months | N/A |
| Peer educator | The peer educator brings the ART directly to the participants’ home. | 3-6 months | N/A |
| Village Health Worker | The participant is able to pick up the ART supply at the village health worker’s home. Village health workers are a trained lay health cadre in rural areas of Lesotho, mainly supporting young mothers as well as HIV and TB clients. In some communities, village health workers are trained and equipped to supply ART. | 3-6 months | N/A |
| Community Adherence Club | Community adherence clubs are formed by people living with HIV from the same village. They regularly meet and discuss health-related topics and take turns in attending the clinic to receive ART. | 3-6 months | N/A |
| Treatment Buddy | A chosen confidant of the participant can pick up the ART at the clinic and bring it to the participant’s home. | 3-6 months | N/A |
| **Component 2: SMS notification options** | | | |
| Adherence reminder | Participant gets notifications to remind her or him to take the medication regularly | 1)Daily  2)Weekly  3)Monthly  Exact weekday and time | 1)Meds time (emoji for clock)  2)Nako ea lithlare (emoji for clock)  3)Recharge!  4)Healthy living!  5)Bophelo bo botle!  6)Me and good health!  7)Nna le bophelo bo botle!  8)Right time!  9)Nake e nepahetseng |
| Refill reminders | Participant gets notified to remind her or him to pick up the medication | 1) 7 days before 2) 3 days before 3) 2 days before 4) 1 day before 5) On the day of ART refill | 1)Visit coming up!  2)Ba Bone uena gheerl / guy  3)GET SOME MORE!  4)Nka tse ling! |
| Viral load result notification | The participant receives a coded message that tells him/her if the result means viral suppression or not. | As soon as the result is available in the laboratory | If suppressed:  1)Happy face emoji 2)Well done, keep it up! 3)Hoooha!! 4)GOT IT!! 5)WOW!!! 6)PELE EA PELE!  If unsuppressed:  1)Keep trying. Do better next time. 2)No leke. Etsa betere ka moso. 3)Ahhh!!! 4)OH NO!!! 5)Battery low. Take action! (neutral face emoji) 6)Battery e tlase. Etsa hohong! (neutral face emoji) |
| **Component 3: Psychosocial support options** | | | |
| By the nurse at the clinic | The nurse at the clinic, who is always there to help with questions and concerns. | According to refill schedule | N/A |
| Saturday Clinic Club | Monthly gathering, on Saturdays at the clinic, of young people living with HIV in the same clinic catchment area. The Saturday Clinic Club is led by the peer educator. They discuss adherence issues and address psychosocial concerns. | monthly | N/A |
| Community Youth Club | The Community Youth Club is similar to the Saturday Clinic Club, but at a central point in the community and includes also HIV-negative youth. Thus, general psychosocial issues are discussed, not HIV/AIDS-focused. | monthly | N/A |
| Phone Call by peer educator | Peer educator calls the participant to see how he/she is doing. | On demand | N/A |
| Home-visit by peer educator | Peer educator visits the participant at home to see, how he/she is doing and facilitate communication within the family. | On demand | N/A |
| School health talk by peer educator | Peer educator comes to the participants school and holds a general health talk, focusing on HIV/AIDS related stigma | On demand | N/A |
| Pitso visit and health talk by peer educator | Pitso is a public village gathering, presided over by the village chief. The peer educator holds a general health talk, focusing on HIV/AIDS related stigma. | On demand | N/A |
| Condom demonstration | Demonstrating and explaining the correct use of a condom to the participant. | Immediately | N/A |
| More information about contraceptives | Refer to responsible nurse to explain the different types of options and their implication with regards to HIV transmission. | Immediately | N/A |
| More information about voluntary male medical circumcision | Refer to responsible nurse to explain what voluntary male medical circumcision is, the benefits and risks and if interested initiate further steps to get an appointment | Immediately | N/A |
| For pregnant women: Linkage to young mothers’ group | Refer to a support group of young mothers who discuss their issues in a confidential space at the clinic | Immediately | N/A |
| For women: Linkage to a female social asset building model | Refer to a group of women who do capacity building for asset-building and entrepreneurship. | Immediately | N/A |
| More information about legal aid and gender-based violence | A flyer is distributed with information and phone numbers to get help | Immediately | N/A |
| No support wanted | N/A | N/A | N/A |

Abbreviations: N/A (not applicable)
