# Supplementary material for: Effectiveness of a peer educator-coordinated preference-based differentiated service delivery model on viral suppression among young people living with HIV in Lesotho: The PEBRA cluster-randomized trial
Source: PLoS Med. 2023 Jan 3;20(1):e1004150. doi: 10.1371/journal.pmed.1004150 (PMC9810159; doi:10.1371/journal.pmed.1004150)
Supplement: S2 Table — (DOCX) [file pmed.1004150.s003.docx]

**Table S2.** Sensitivity analysis with adjustment for baseline viral load

|  | **Total** | **VL<20 c/mL** | **VL>=20 c/mL or missing** | **Adjusted odds ratio**  **(95% CI)** | **p-value** |
| --- | --- | --- | --- | --- | --- |
| ***Primary endpoint analysis model*** |  |  |  |  |  |
| n | N=307 | n=194 | n=113 |  |  |
| Arm allocation |  |  |  |  |  |
| Control | 157 (51%) | 95 (61%) | 62 (55%) | 1 |  |
| Intervention | 150 (49%) | 99 (66%) | 51 (45%) | 1.27 (0.79 to 2.03) | 0.327 |
| District |  |  |  |  |  |
| Butha-Buthe | 145 (47%) | 100 (52%) | 45 (40%) | 1 |  |
| Leribe | 39 (13%) | 24 (12%) | 15 (13%) | 0.71 (0.34 to 1.49) | 0.370 |
| Mokhotlong | 123 (40%) | 70 (36%) | 53 (47%) | 0.59 (0.36 to 0.98) | 0.040 |
| Sex |  |  |  |  |  |
| Female | 218 (71%) | 136 (70%) | 82 (73%) | 1 |  |
| Male | 89 (29%) | 58 (30%) | 31 (27%) | 1.11 (0.66 to 1.88) | 0.688 |
| ***Primary endpoint analysis model, adjusted for baseline VL*** [1] |  |  |  |  |  |
| n | n=273 | n=175 | n=98 |  |  |
| Arm allocation |  |  |  |  |  |
| Control | 140 (51%) | 85 (48%) | 55 (56%) | 1 |  |
| Intervention | 133 (49%) | 90 (51%) | 43 (44%) | 1.31 (0.79 to 2.18) | 0.298 |
| District |  |  |  |  |  |
| Butha-Buthe | 126 (46%) | 89 (51%) | 37 (38%) | 1 |  |
| Leribe | 35 (13%) | 21 (12%) | 14 (14%) | 0.57 (0.26 to 1.26) |  |
| Mokhotlong | 112 (41%) | 65 (37%) | 47 (48%) | 0.65 (0.38 to 1.13) | 0.131 |
| Sex |  |  |  |  |  |
| Female | 192 (70%) | 121 (69%) | 71 (72%) | 1 |  |
| Male | 81 (30%) | 54 (31%) | 27 (28%) | 1.28 (0.72 to 2.26) | 0.396 |
| Baseline VL category |  |  |  |  |  |
| <20 c/mL | 166 (61%) | 117 (67%) | 49 (50%) | 1 |  |
| >=20 c/mL | 107 (39%) | 58 (33%) | 49 (50%) | 0.50 (0.29 to 0.85) | 0.010 |

Abbreviations: CI (confidence interval), VL (viral load)

[1] excluding those with missing baseline VL
