# Supplementary material for: Effectiveness of a peer educator-coordinated preference-based differentiated service delivery model on viral suppression among young people living with HIV in Lesotho: The PEBRA cluster-randomized trial
Source: PLoS Med. 2023 Jan 3;20(1):e1004150. doi: 10.1371/journal.pmed.1004150 (PMC9810159; doi:10.1371/journal.pmed.1004150)
Supplement: S3 Table — (DOCX) [file pmed.1004150.s004.docx]

**Table S3.** Sensitivity analyses 2 and 3 on primary endpoint

|  | **Total**  **(n=307)** | **Control (n=157)** | **Intervention (n=150)** | **Adjusted odds ratio (95% CI)** | **p-value** |
| --- | --- | --- | --- | --- | --- |
| ***Wider endpoint window: VLs up to 90 days past protocol-defined window*** |  |  |  |  |  |
| VL <20 copies/mL | 197 (63%) | 96 (61%) | 101 (67%) | 1.29 (0.8 to 2.07) | 0.292 |
| ***Per protocol: participants who attended both the 6-month and 12-month visit*** |  |  |  |  |  |
| VL <20 copies/mL | 183 (60%) | 88 (56%) | 95 (63%) | 1.33 (0.84 to 2.12) | 0.222 |

Abbreviations: CI (confidence interval), VL (viral load)
