# Supplementary material for: Effectiveness of a peer educator-coordinated preference-based differentiated service delivery model on viral suppression among young people living with HIV in Lesotho: The PEBRA cluster-randomized trial
Source: PLoS Med. 2023 Jan 3;20(1):e1004150. doi: 10.1371/journal.pmed.1004150 (PMC9810159; doi:10.1371/journal.pmed.1004150)
Supplement: S4 Table — (DOCX) [file pmed.1004150.s005.docx]

**Table S4.** Subgroup analyses on primary endpoint

|  | **VL <20 copies/mL at 12 months** | | | **p-value interaction** |
| --- | --- | --- | --- | --- |
|  | **Total**  **(n=307)** | **Control (n=157)** | **Intervention**  **(n=150)** |  |
| ***Age groups*** |  |  |  |  |
| 15-19 | 168 | 49/76 (64%) | 66/92 (72%) | - |
| 20-24 | 139 | 46/81 (57%) | 33/58 (57%) | 0.515 |
| ***Sex*** |  |  |  |  |
| female | 218 | 70/119 (59%) | 66/99 (67%) | - |
| male | 89 | 25/38 (66%) | 33/51 (65%) | 0.410 |
| ***Marital status*** |  |  |  |  |
| separated or divorced or widowed | 8 | 2/4 (50%) | 3/4 (75%) | - |
| single | 206 | 63/99 (64%) | 73/107 (68%) | 0.579 |
| married | 93 | 30/54 (56%) | 23/39 (59%) | 0.532 |
| ***Occupational status*** |  |  |  |  |
| (self-)employed | 22 | 6/9 (67%) | 7/13 (54%) | - |
| Attending school | 93 | 25/36 (69%) | 42/57 (74%) | 0.481 |
| None of the above | 192 | 64/112 (57%) | 50/80 (63%) | 0.404 |
| ***Time of ART exposure*** |  |  |  |  |
| <1 year | 53 | 20/36 (56%) | 7/17 (42%) | - |
| 1-5 years | 151 | 49/82 (60%) | 53/69 (77%) | 0.123 |
| 6-10 years | 64 | 15/23 (65%) | 28/41 (68%) | 0.577 |
| >10 years | 39 | 11/16 (69%) | 11/23 (48%) | 0.621 |
| ***On a DTG-based regimen at time of primary endpoint*** |  |  |  |  |
| Yes | 268 | 93/141 (66%) | 90/127 (71%) | - |
| No | 39 | 2/16 (13%) | 9/23 (39%) | 0.208 |

No significant interaction terms (p-interaction) were found.

Abbreviations: ART (antiretroviral therapy), DTG (dolutegravir), CI (confidence interval), VL (viral load)
