# Supplementary material for: Effectiveness of a peer educator-coordinated preference-based differentiated service delivery model on viral suppression among young people living with HIV in Lesotho: The PEBRA cluster-randomized trial
Source: PLoS Med. 2023 Jan 3;20(1):e1004150. doi: 10.1371/journal.pmed.1004150 (PMC9810159; doi:10.1371/journal.pmed.1004150)
Supplement: Attachment — Submitted filename: PEBRA_response_to_reviewers_20221012.docx [file pmed.1004150.s006.docx]

**Response to Reviewers**

*Effectiveness of a peer educator-coordinated preference-based differentiated delivery model on viral suppression among young people living with HIV in Lesotho: The PEBRA cluster randomized trial*

**Requests from the Editor:**

1. Author summary - At this stage, we ask that you reformat your non-technical Author Summary.

***Response:*** *We added the Author Summary.*

Abstract:

Please report your abstract according to CONSORT for abstracts, following the PLOS Medicine abstract structure (Background, Methods and Findings, Conclusions)

***Response:*** *We adapted accordingly.*

2. Please rename “Introduction” as “Background.”

***Response:*** *We adapted accordingly.*

3. Background: final section should clearly state what you were comparing across the two delivery models

***Response:*** *We modified the last 2 sentences in the Background and the first paragraph in the Methods section to clarify:*

Southern and Eastern Africa is home to more than 2.1 million young people aged 15-24 years living with HIV. As compared with other age groups, this population group has poorer outcomes along the HIV care cascade. Young people living with HIV and the research team co-created the PEBRA (Peer Educator-Based Refill of ART) care model. In PEBRA a peer educator delivered services as per regularly assessed patient preferences for medication pick-up, SMS notifications and psychosocial support. The cluster-randomized trial compared PEBRA model versus standard clinic care (no peer educator and ART refill done by nurses) in three districts in Lesotho.

Individuals taking antiretroviral therapy aged 15-24 years at 20 clinics (clusters) were eligible. In the ten clinics randomized to the intervention arm, participants were offered the PEBRA model, coordinated by a trained peer educator and supported by an eHealth application. In the ten control clusters, participants received standard nurse-coordinated care without any service coordination by a peer educator. The primary endpoint was 12-month viral suppression below 20 copies/mL. Analyses were intention-to-treat.

4. Please provide the number of clinics in each arm (i.e., control and intervention groups)

***Response:*** *There were ten clinics per group. We added the information to the Methods section.*

5. Please quantify the main results (with p values in addition to 95% CI).

***Response:*** *We added p-values to all results. Also, see answer to question number 4, reviewer #1, for more details.*

6. Please provide the number of participants lost to follow up in each group

***Response:*** *We provided the number of lost to follow by group.*

7. Please include a summary of adverse events if these were assessed in the study.

***Response:*** *We did not systematically assess adverse events since the PEBRA trial was a pragmatic implementation trial, not investigating any experimental medication, only well-established ART, following the Lesotho national guidelines.*

8. In the last sentence of the Abstract Methods and Findings section, please describe the main limitation(s) of the study's methodology.

***Response:*** *We included the main limitation in one sentence to keep it brief:*

The main limitation was that the data collectors in the control clusters were also young peers, who used the same data collection tool, however, they used a restricted version of the PEBRApp and thus were not able to provide the PEBRA model.

Main text:

9. Please rename the Introduction as “Background.”

***Response:*** *We adapted accordingly.*

10. Background: Please describe past research and explain the need for and potential importance of your study. Indicate whether your study is novel and how you determined that. For example, if there has been a systematic review of the evidence related to your study (or you have conducted one), please refer to and reference that review and indicate whether it supports the need for your study.

***Response:*** *We added more background information, including the systematic reviews we found on the topic and clarified the rationale for the PEBRA trial:*

Several systematic reviews concluded that evidence for interventions to improve engagement in HIV care among adolescents and young adults low-resource settings is of low quality (ref) and in general DSD models are not designed for young people nor led by young people(ref). The Zvandiri trial in Zimbabwe demonstrated that adolescents benefit from community adolescent treatment supporters (ref), however a peer educator coordinated service delivery at the facility has not been evaluated yet.

11. Introduction: Please conclude the introduction with a clear description of the study question or hypothesis including the outcomes of interest.

***Response:*** *We added the primary hypothesis at the end of the introduction/background:*

The PEBRA cluster-randomized trial compared the PEBRA model versus standard clinic care, where no peer educator coordinated care was offered, in three districts in Lesotho. We hypothesized that PEBRA will have a beneficial effect on viral suppression and other clinical and HIV care-related outcomes.

12. Methods: Please clearly state how many clusters were in each arm

***Response:*** *Under “Statistical analysis” we mentioned how many clusters were planned for the target sample size. In the third sentence in the “Results” section we clarified that randomization of the 20 health facilities was 1:1 and Figure 2 (CONSORT flow chart) indicates that there were ten cluster per arm.*

13. Under outcomes, please define what is meant by “loss to follow-up”

***Response:*** *We defined participants loss to follow-up as more than 2 months late for a scheduled visit or medication pick-up and no information found about the participant. We added the definition under outcomes. More details about the outcomes are provided in the published study protocol. We also added this information at the end of the section Outcomes.*

14. Please complete the “CONSORT extension for Cluster Trials” and ensure that all components of CONSORT are present in the manuscript and include definition of adherence. When completing the checklist, please use section and paragraph numbers, rather than page numbers.

***Response:*** *We have completed the CONSORT extension for Cluster Trials checklist, referenced it and uploaded it:*

The PEBRA trial is registered with ClinicalTrials.gov (<https://clinicaltrials.gov/ct2/show/NCT03969030>; prospectively registered on May 31, 2019), and reported as per the CONSORT extension for Cluster Trials guidelines (S1 Checklist).

15. Please add the following statement, or similar, to the Methods: "This study is reported as per the “CONSORT extension for Cluster Trials” guideline (S1 Checklist)."

***Response:*** *We have included the statement in the Methods (Ethics section):*

The PEBRA trial is registered with ClinicalTrials.gov (<https://clinicaltrials.gov/ct2/show/NCT03969030>; prospectively registered on May 31, 2019), and reported as per the CONSORT extension for Cluster Trials guidelines (S1 Checklist).

16. Please clarify whether parental consent was sought for participants aged less than 18 years.

***Response:*** *Our research included adolescents and young people living with HIV from the age of 15 years to 24 years old, a key group that is often neglected in research projects. One reason is that the consent process for people below 18 years is more complicated and usually requires a caregiver. This leads to decreased feasibility (i.e., most young people do not come with their caregivers to the health facility) and to a bias in the study (i.e., caregivers not consenting although the participants would agree). PEBRA trial was an implementation trial, that did not test any new medication or experimental substances but tried to tailor the services more accordingly to the preferences of the young people with the help of peer educators. We thus strongly felt that it is justifiable and important that these young people give consent without parental consent, as it is being discussed in other research projects (references 1-5 in box below).*

**References**

1) Singh JA, Karim SSA, Karim QA, et al. Enrolling Adolescents in Research on HIV and Other Sensitive Issues: Lessons from South Africa. PLOS Med 2006;; 3: e180.

2) Gilbert AL, Knopf AS, Fortenberry JD, Hosek SG, Kapogiannis BG, Zimet GD. Adolescent Self-ˇConsent for Biomedical HIV Prevention Research. J Adolesc Health Off Publ Soc Adolesc Med 2015;; 57: 113–9.

3) Fisher CB, Arbeit MR, Dumont MS, Macapagal K, Mustanski B. Self-ˇConsent for HIV Prevention Research Involving Sexual and Gender Minority Youth: Reducing Barriers Through Evidence-ˇBased Ethics. J Empir Res Hum Res Ethics 2016;; 11: 3–14.

4) Nelson RM, Lewis LL, Struble K, Wood SF. Ethical and Regulatory Considerations for the Inclusion of Adolescents in HIV Biomedical Prevention Research. JAIDS J Acquir Immune Defic Syndr 2010;; 54: S18.

5) Pomfret S, Karim QA, Benatar SR. Inclusion of Adolescent Women in Microbicide Trials: A Public Health Imperative! Public Health Ethics 2010;; 3: 39–50.

*Therefore, we asked and discussed with the ethic committee to waive parental consent for the 15-17 years old study participants, which they approved. Find the communication letter uploaded separately (“PEBRA study_EC LS cover letter_24.05.19”) as well as the corresponding ethics approval (“PEBRA_EC LS approval_June19”). It was described in detail in the published study protocol and we included the information in the current manuscript in section “Ethics statement”:*

The trained study staff obtained the individual written informed consent from the participants before inclusion into the PEBRA trial. To minimize selection bias, the ethics committees agreed to waive parental consent for the 15-17 years old study participants as outlined in the approved PEBRA study protocol (ref). Illiterate study participants provided a thumbprint and a literate witness (independent to the trial and chosen by the participant) co-signed the form. The informed consent was provided in the local language, Sesotho, and the participant received a copy of the consent form.

17. Please provide definitions for all abbreviations used in tables and figures and include these as footnotes, e.g., ART, IQR, QoL

***Response:*** *We added the definitions for all abbreviations in all tables and figures*

18. Results: Please clearly state the number of clusters per arm.

***Response:*** *We adapted accordingly.*

19. In the main text and tables, please provide both 95% CIs and p values, where appropriate

***Response:*** *We added p-values to all results. Also, see answer to question/comment number 4, reviewer #1.*

20. The terms gender and sex are not interchangeable (as discussed in <https://scanmail.trustwave.com/?c=22737&d=lriI45_TyB9n7P5n-w2RQa7tD1_Pt6UXuNeb8_ulZQ&u=https%3a%2f%2fwww%2ewho%2eint%2fhealth-topics/gender> ); please use the appropriate term.

***Response:*** *Thank you for spotting this important inconsistency. We adapted accordingly and changed gender to sex.*

21. Please remove the “Competing of Interests” statement at the end of the main text. This information is captured in the metadata obtained in the submission form

***Response:*** *We removed the Competing of Interests statement.*

**Reviewer #1:**

1. This paper reports a cluster randomised trial evaluating a peer-led intervention for improving viral suppression amongst young people with HIV. Generally, the statistical methods and reporting was good. I have some comments below

Abstract: The authors report the primary outcome and a couple of the secondary outcomes. I would recommend adding a statement about other secondaries e.g. 'There were no significant differences between arms in other secondary outcomes', or just report the primary outcome in the abstract. At the moment, it appears selective to report one secondary outcome that had a significant difference.

***Response:*** *Thank you for your positive feedback. We added your suggestion ('There were no significant differences between arms in other secondary outcomes')* *to the results section of the abstract.*

2. Methods, randomisation: how was the randomisation stratified, using one block in each strata?

***Response:*** *Yes, randomization was stratified by district, using one block of eligible clinic-clusters per district, and one randomization event per district was held. At these events, health facility representatives drew opaque, sealed, equally sized envelopes containing the group allocation (control or intervention) from a Mokorotlo (traditional Lesotho hat), and disclosure took place only once all facilities had drawn their envelope. Additionally, the sequence of drawing was randomly selected in advance by an independent person drawing from a second pile of opaque, sealed envelopes containing the names of the facility. Clinics were assigned (1:1) to either offer standard of care or the PEBRA model. We added a brief sentence to further clarify the randomization:*

Randomization was stratified by district (Butha-Buthe versus Leribe versus Mokhotlong), using one block per strata.

3. Statistical analysis: for the sample size calculation, the type I error rate is not given.

***Response:*** *We mentioned the type 1 error in the published study protocol, but forgot to mention it here. We added it.*

4. Statistical analysis, line 225 "(estimated using the delta method)"  - I am not sure this is right - the logistic regression gives the confidence interval for odds-ratios.

***Response:*** *We initially used the SE for the odds ratio - derived using the delta method -to determine a symmetric approximation for the 95% CI. Following this comment and your suggestion to add p-values of the regression coefficients below, we now feel that it makes more sense to replace the CIs by the Wald CIs of the regression coefficients. We adapted all analyses (95% CI and p-values) accordingly.*

5. Statistical analysis: could more be added about how missing data was handled for secondary outcomes?

***Response:*** *Thank you for spotting this missing information. For the primary endpoint it is mentioned, but not for the secondary endpoints. Different to the primary endpoint, for all secondary endpoints we considered complete case only. We added this information to the statistical analysis section:*

For the secondary endpoints, we used the complete case set.

6. Results - I would recommend that p-values are given alongside CIs in the tables, as the trial was powered for hypothesis testing.

***Response:*** *We added the p-values.*

**Reviewer #2:**

1. This paper describes a very well conducted RCT. It is clearly described and methods are sound. It approaches an extremely important question - how to improve VL suppression amongst adolescents and young people in Sub-Saharan Africa. The trial did (primarily) not produce significant results, but that does not make it any less publishable - in contrast, I think it is important that we do publish these studies, so that we can understand more about what doesn't and does work for this group. Minor comments

It would be helpful to give a bit more information about the intervention

***Response:*** *Thank you for this encouraging feedback. We agree that the information presented was little and referring back to the published protocol is not ideal and inconvenient. We added an additional supplement (Supplementary Table 1) that outlines each intervention component and its options in detail. An analysis of each intervention component and preferences over time will follow in a secondary analysis of the trial data (currently in review at a sister journal of the PLOS Med).*

2. in Table 1, it may be good to check on the terms used 'gay/ lesbian/straight' - it's not my area but plenty of people can give you advice on the best terms to use for this

***Response:*** *We changed the terms of sexual orientation to homosexual and heterosexual.*

3. from my reading (may be incorrect) both groups improved in terms of VL suppression. I do wonder whether there's a Hawthorne effect here, particularly with the attention given to participants by peer data collectors in the control group. Perhaps just having someone pay attention to them was helpful. I think there is value to saying that the groups both improved (if I read Table 1 correctly)

***Response:*** *We agree with the reviewers’ interpretation. We mentioned this point under limitations (last limitation), but have emphasized the improvement in both groups in the discussion section, second paragraph, and also rephrased our interpretation of the uncertainty regarding the primary endpoint:*

Overall, viral suppression increased from 166/307 (54%) at baseline to 194/307 (63%) within 12 months of follow-up. However, there was no conclusive evidence of a significantly higher rate of viral suppression at 12 months among participants in the intervention clusters compared to standard of care.

4. A very thoughtful discussion and very interesting that the peer model may work best for adolescents rather than the young people age group. I might suggest adding that pre-specified effect modification analysis into the abstract as it may be important (was it under-powered?)

***Response:*** *This treatment effect modifier is very likely underpowered and no significant interaction terms (p-interaction) were found. The trial was unfortunately not powered to detect any differences in subgroups. Therefore, we suggest not adding more emphasize on this analysis.*

5. Finally, well done to the authors on doing this within a CIPHER grant (which is not a lot of money for an RCT). I would absolutely recommend publishing this.

***Response:*** *Thank you very much.*

**Reviewer #3:**

1. Many thanks for submitted this manuscript to PLOS. This is an important areas of study given the relatively low rates of VLS amongst adolescents. There are a few issues that I wish to raise for your consideration in revising this manuscript.

Why were rural districts/facilities chosen? Would there have been any difference in your results in urban areas were chosen?

***Response:*** *Thank you for your positive feedback. The districts our research consortium is active (Butha-Buthe, Mokhotlong and Leribe districts) are mostly rural; 70% of the overall population in Lesotho lives in rural settlements. The urban population is mostly served by the district hospitals. The district hospitals provide fewer uniform services, would have been a logistical challenge to incorporate in our model of care, and HIV care outcomes (e.g. viral suppression) among urban population are generally better than among the rural population. Moreover, our research collaboration is stronger with the nurse-led clinics than the hospitals and a successful intervention would be easier to scale up across the clinics than the hospitals. All these reasons led to the decision to conduct the study at the rural health clinics. We added these thoughts to our section “Study design and participants”:*

We decided to conduct this trial only across nurse-led clinics in rural areas because these are the most common health facilities across Lesotho, they provide standardized services (e.g., important for the control arm comparison) and the population they serve reports generally worse HIV outcomes compared to the population served at the district hospitals.(ref)

2. Was care provided by public facilities different (especially in the control arm) compared to the missionary led facilities?

***Response:*** *Thank you for bringing up this important point.* *Care provided across the nurse-led clinics, public or missionary, are very similar. The main difference is that missionary-based clinics provide restricted sexual and reproductive health services. However, sexual and reproductive health services (e.g. VMMC referral and condom demonstration) is only a small part of the PEBRA intervention component. Moreover, of the 20 participating clinics, 5 were missionary-based and by chance 3 were allocated to control and 2 to intervention arm. We added this point to the first paragraph in the Results section:*

Three clinics in the control arm and two in the intervention arm were missionary facilities.

3. It is now well known that designing interventions for adolescents younger than 19 (more of whom are presumable still in school and living with care givers) differently to those 20 years and older should be considered - did you consider this? if not why not?

***Response:*** *From our viral load database we knew that the key population of “Youth” (as per WHO definition aged 15 – 24 years old) has the worst virologic outcomes compared to other age groups and thus we wanted to include and design an intervention that includes not only adolescents but also young adults. The entire idea behind this preference-based differentiated model was that services are adapted regularly by the peer educator regularly assessing the preferences and adapting accordingly. The only common pillar was that the model was delivered by a peer. The PEBRA model was developed in close collaboration with peer educators, young people living with HIV and youth activists. The involvement of the peer educator was suggested and welcomed by everyone, also for young adults. However, it was important to the group that individual preferences are regularly assessed, and one concern was exactly that the older participants may have different preferences than the younger ones. Thus, we included a pre-specified treatment effect modification analyses by age – which indicated exactly what the reviewer already pointed out (less effect among older participants), but it was likely underpowered. In a second paper, we will outline in detail the preferences among the intervention participants and how these preferences changed over time and by age. This second paper is currently under review at a sister paper of the PLOS Medicine. We would like to refer to our discussion where we discuss this subgroup analysis.*

4. Were the activities in the intervention arm co-designed by young people or did the researchers assume that they will make a difference?

***Response:*** *The PEBRA model was developed in close collaboration with peer educators, young people living with HIV, youth activists, clinicians, district Ministry of Health authorities, Sentebale youth leaders, SolidarMed and Swiss Tropical and Public Health Institute research staff as well as local and international IT developers. During a series of workshops, these stakeholders defined the current main challenges in adolescent HIV care in Lesotho, possible strategies within the PEBRA model to overcome these challenges building on existing resources at the study health facilities, the role of the peer educator within the PEBRA model, and the scope and design of the eHealth tablet-based application that was specifically developed for the PEBRA trial. We summarized the process of development of the PEBRA model in the third paragraph under section “Intervention and Control”.*

**Reviewer #4:**

1. This is an interesting study on a topic of high importance in the HIV field. Unfortunately, the results did not show a statistically significant impact for the primary outcome. Regardless, I think it is important to report studies like this in high impact journals. That being said, I think there are a number of issues which, if addressed, would substantially improve the paper.

***Response:*** *Thank you for your constructive and positive feedback.*

2. Abstract, in the Conclusions, I think "was associated with" rather than "led" may be a more appropriate interpretation.

***Response:*** *Thank you for pointing out this important nuance. We acknowledge that this result should be interpreted with caution. We therefore dropped it and rephrased the conclusion:*

Preference-based peer-coordinated care for young people living with HIV, compared to nurse-based care only, did not lead to conclusive evidence for an effect on viral suppression.

3. Methods, some more detail on how recruitment was done would be helpful, e.g. was it a convenience sample of whomever showed up at clinic during that study period, etc.?

***Response:*** *After the clinics were randomized at the public randomization event, the peer educators were trained and then started to screen at the same time at all 20 health facilities on a rolling basis, i.e., a convenience sample. The peer educators were instructed to recruit every day (Monday-Saturday) and include every young adult that shows up at the clinic until a minimum sample size per clinic is reached. Thye received a screening log and noted exclusion criteria (4 participants per arm were screened out, see figure 2). Importantly, all peer educators recruited in the same time period and finished recruitment quickly (3 months). With these measures we tried to minimize selection bias in this cluster-randomized trial. We added some more details in the section “Procedures”:*

During the recruitment period, the PE actively screened all young people attending their health facility for inclusion on a rolling basis and obtained informed consent. The PEs recruited from Monday to Saturday on a strictly rolling basis and followed a screening log file. Recruitment happened at all 20 health facilities concurrently.

4. Results, how many young people were screened out and not even approached about possible enrollment in the study?

***Response:*** *8 participants in total were screened out (4 per arm), all because they declined participation (figure 2). Although we may have expected a few more reasons for screened out (e.g. due to age), it overall makes sense since the eligible criteria were very broad: Everyone attending care at a participating facility, taking ART, being 15-24 years old and providing written informed consent. We added the number of people screened out per group to the first paragraph of the “Results” section:*

Between November 6, 2019, and February 4, 2020, 315 young people (aged 15-24 years) living with HIV and receiving ART care at the 20 clusters were identified and approached. Of these, 8 (2.5%) declined enrolment, 4 in each group.

5. Methods, The role of the app was not exactly clear. Was this app just on the peer's phone? Did participants use the app?

***Response:*** *Yes, this app was an android-based application, installed on the tablet of each peer educator. The participants did not use the PEBRApp. The initial idea was to create also a PEBRApp version for the participants (e.g. to use as messenger app to communicate between peer educator and participant). But during the series of workshops about the intervention and the design of the app, the consensus was that too few participants would have regular access to a smartphone in rural Lesotho. Therefore, we restricted the PEBRApp and the communication channel to SMS technology and implemented SMS notifications instead of app-based notifications. SMS notifications were sent out automatically by the PEBRApp and always included a free-of-charge call-back option to the PE’s phone number. As such the PEBRApp helped the PE not only to collect data, regularly assess the preferences of their peers and to keep track of the ART refill and next assessment dates, but also ensured regular contact between the PE and the participant. Some of this information is provided in the published study protocol but we agree with the reviewer to elaborate in more detail and thus adapted the second and third paragraph in section “Intervention and control”:*

This process was guided by the PEBRApp, an android-based application, installed on the tablet of the PEs. The PEBRA model and the PEBRApp were developed in close collaboration with peer educators, young people living with HIV, youth activists, clinicians, district Ministry of Health authorities, Sentebale youth leaders, SolidarMed and Swiss Tropical and Public Health Institute research staff as well as local and international IT developers. During a series of workshops, these stakeholders defined the current main challenges in adolescent HIV care in Lesotho, possible strategies within the PEBRA model to overcome these challenges building on existing resources at the study health facilities, the role of the PE within the PEBRA model, and the scope and design of the PEBRApp.

Due to limited access to smartphones in rural Lesotho, the consensus from the formative workshop series was to build an application for the PEs only, with a communication channel between PE and participants based on SMS technology. As such the PEBRApp helped the PE not only to collect data, regularly assess the preferences of their peers and to keep track of the ART refill and next assessment dates, but also ensured regular contact between the PE and the participant. More details and screenshots about the PEBRApp are provided in the published study protocol and the code is open-source on github (<https://github.com/chrisly-bear/PEBRApp>). Any selected SMS notifications were sent out automatically according to chosen frequency and content and always included a free-of-charge call-back option to the PE’s phone number.

6. Methods, were there no peer educators at all in the control arm?

***Response:*** *there were also young peer educators in the control arms who used the same data collection tool, i.e., a restricted version of the PEBRApp. They were however not involved in the coordination of care, were not trained in the PEBRA model and all its components and the PEBRApp offered only very limited features (i.e. collecting endpoints). However, their sole presence and interaction with the participants, of course, may have had an influence. We addressed this point in our discussion section, under limitations, number four: “*Fourth, the data assessors in the control clusters were also young peers, who used the same data collection tool (a restricted version of the PEBRApp). Although they were specifically instructed and were not able to provide the PEBRA model, their presence may have positively influenced follow-up of participants at control clinics.”

7. Methods, were study participants compensated?

***Response:*** *Study participants were not compensated. We added this information in the Methods, section “Ethics statement”.*

8. Methods/Results. There was no substantial process data presented which greatly limits interpretation of this study. For example, who actually received the DSD services? What did they choose? How often did they change? How much, if any, did the peers interact with the patients? Without knowing how well the intervention was implemented, it is difficult to draw conclusions.

***Response:*** *We acknowledge that these questions are of high importance. We longitudinally captured and analyzed a large amount of DSD preference data of the intervention arm (preferences for each intervention component including changes over time). We felt that it would be overwhelming for the reader if this was additionally presented in the current manuscript. We thus submitted the DSD preference analysis as a separate paper to a sister journal of the PLOS Med (after rejection by PLOS Med). Moreover, the idea of the main manuscript was to present the overall treatment effect such a preference-based model of care will have, irrespective of strictly adhering to the chosen DSD components. Importantly, the PEBRA model was successfully implemented in all intervention clinics and all participants accessed the model but the detailed services chosen varied based on preferences.*

*However, we added this point as a limitation in our discussion:*

Fourth, the study design and statistical power did not allow for the evaluation of the effectiveness of each individual feature of the PEBRA model, but a descriptive analysis of longitudinal preference data of each component is planned as a follow-up manuscript.

9. Results, there seems to be a potentially significant difference in age between arms, was adjusting for this considered? The number of children may also be an important difference that might have impacted results.

***Response:*** *As per our statistical analysis plan, we did foresee adjustment for clinically important random imbalanced factors. After discussion with the study team (clinicians, statisticians, researchers), we decided to adjust for sex. We considered age as not being randomly imbalanced enough and had age as a pre-planned treatment effect modifier. Number of children was considered not clinically important enough although quite imbalanced. Upon request of the reviewer, we added this variable as a post hoc analysis to the primary model and it yielded similar results. We described the procedure of adjustment variable selection in the second paragraph in section “Statistical analysis”.*

10. Discussion, is transferring out of care necessarily a bad thing? This seems to be implied throughout, but what evidence is there for this?

***Response:*** *But we agree with the reviewer that this should be interpreted with caution. We removed the judgement in the second paragraph of the Discussion and adapted the Conclusion:*

This trial is one of few randomised clinical trials focusing on differentiated service delivery among young people living with HIV. During the study period of the PEBRA trial viral suppression rates in both groups increased by 9%. Preference-based peer-coordinated care for young people living with HIV, compared to nurse-based care only, did not lead to conclusive evidence for an effect on viral suppression. More large-scale research is needed to understand the effect of peer-led models of care among the youth.

11. Results, Table 2 has no p values. There are potentially good reasons for this but I am not sure what the journals standards are for this.

***Response:*** *We added p-values to all results. Also, see answer to question/comment number 4, reviewer #1.*

12. Conclusions, it is not clear that the peers were "feasible" because no real feasibility data were presented.

***Response:*** *Although our study team was in regular contact with the peer educators and the participating health facilities and received overwhelming positive feedback and only few challenges with involving peer educators at the facility level in the coordination of care for their peers, we agree that we did not collect and present real feasibility indicators. Thus, we adapted the conclusion and removed this point (see point 10).*

13. Conclusions, not sure I would say this study "supports WHO's recommendation". There are some suggestions of benefit but the primary outcome was negative

***Response:*** *We agree with the reviewer that our wording was too strong. However, we disagree that the primary outcome was negative (the intervention group showed 5 percentage points higher viral suppression), but suggest interpreting the confidence intervals as inconclusive evidence of effect. Thus, we adapted the conclusion and removed this point (see point 10).*

14. Conclusions, qualitative findings would be helpful to understand potential benefits not captured by the quantitative results. Was this considered?

***Response:*** *We fully agree with the reviewer that more qualitative insights are crucial to understand the mechanisms of action and dynamics of preferences better. In a second paper we analyzed qualitative and semi-qualitative data to present longitudinal change of preferences over the study period. This paper is currently under review at a sister journal of the PLOS Medicine.*

**Reviewer #5:**

INTRO

1. “In Lesotho, similar to other countries in the region, HIV care is hardly adapted to adolescents and young adults and young peers only play a limited role in providing services.”- I would tone this statement down a bit, because “hardly adapted” is a strong term that does not quite reflect that there do exist adaptations for AYA HIV care in the African region. E.g. Zvandiri, Operation Triple Zero, Teen Clubs (see WHO technical report on “Adolescent-Friendly Health services for Adolescents Living with HIV: from theory to practice.” <https://scanmail.trustwave.com/?c=22737&d=i-mp41IuQ8uRLIAwuCBqSq69KHkUpVR_nm3s57W6w&u=https%3a%2f%2fwww%2ewho%2eint%2fpublications%2fi%2fitem%2fadolescent-friendly-health-services-for-adolescents-living-with-hiv>) This report summarizes many of the DSDs available for adolescents especially in African countries.

**Response:** *Thank you for your review and raising this point. We agree; we revised the language, added the reference provded by the reviewer, and provided a more specific rationale of the focus of this trial (see also comment 10 of the Editor):*

In Lesotho, similar to other countries in the region, efforts to adapt HIV care to adolescents and young adults are promoted (ref), but young peers still only play a limited role in providing services.

Several systematic reviews concluded that evidence for interventions to improve engagement in HIV care among adolescents and young adults low-resource settings is of low quality (ref) and in general DSD models are not designed for young people nor led by young people(ref). The Zvandiri trial in Zimbabwe demonstrated that adolescents benefit from community adolescent treatment supporters (ref), however a peer educator coordinated service delivery at the facility has not been evaluated yet.

METHODS

2. “Young people were eligible for inclusion if they were living with HIV, registered for HIV care at one of the eligible clusters, aged 15–24 years, taking ART and able to provide informed consent.” Please include the age of consent for participation as is relevant for this study in Lesotho.

**Response:** *Thank you for this important clarification. Age of consent for HIV testing in Lesotho is 12 years, age of ART start at 16 years and general research consent at 18 years. Therefore, we specifically applied for a waiver of parental consent for participants aged 15-18 years of age. We explain this in detail in comment/question number 16 of the Editor.*

3. “Third, the PE systematically assessed the feasibility of the options chosen, as not all options are available to everyone all the time…” I expect that feasibility or availability would affect the preference and the outcomes given that not all participants got their preferences at the times they selected them? It would be good to provide this caveat and speak to it perhaps as a non-modifiable limitation.

**Response:** *We agree with the reviewer that this is an important point. We have analysed this data in detail and are about to publish it as a secondary manuscript (under review at a sister journal of PLOS Med) and added a limitation. We kindly refer to the detailed answer given to question/comment number 8 of Reviewer #4.*

4. By the middle of the narrative on the Intervention and Control description, one notices that there is no clear statement of who is using the PEBRA app. Who exactly was the intended user of the PEBRAApp? Was it only the PE? Did the rural patients use the app too, because that would be a problem especially if the patients were not formally educated or literate enough or did not have access to the needed smart devices and phones and Internet data to use the app.

**Response:** *The PEBRApp was intended for the peer educators only. We kindly refer to the answer of question/comment number 5 of Reviewer #4.*

5. “These service preferences were assessed at enrolment and thereafter every month for participants with unsuppressed VL (> 1000 copies/mL)” this should read greater than or equal to (≥) 1,000.

**Response:** *Thank you for spotting this typo. This was corrected accordingly.*

6. Typo/omission: “At health facilities randomized to the control arm, the study participants received the standard of care offered at nurse-led rural health facilities in Lesotho, i.e. ART refill at the clinic **and** nurse-led support.” Also, please give a brief description of what nurse-led support entailed in the standard of care.

**Response:** *We modified accordingly. We summarized the care provided in control in figure 1. We added the reference to figure 1 at the end of the sentence.*

7. Data collection section: “Data were collected” .by whom? Please indicate exactly who did what in the data collection section, so that it will be clear where the PEs were doing data collection and where routine program staff or other study staff were doing data collection.

**Response:** *The statement was adapted and rephrased accordingly:*

Data were collected by the PEs in the intervention arm and trained young study staff members using the password protected PEBRApp in both arms. However, the PEBRApp in control arm was limited to baseline and follow-up data collection and thus did not allow any differentiated service delivery.

8. The “satisfaction with care” questionnaire – please put that term in quotes or capitalize the first S and C, as it is the name of a document.

**Response:** *We adapted accordingly.*

9. The primary outcome was viral suppression at 12 months, defined as the proportion of all
10. “…participants in care with a VL below 20 copies/mL ***at*** 12 months (range 9-15 months) after enrolment.”

**Response:** *We adapted accordingly.*

11. “For the control arm, based on the same routine cohort data we assumed a viral suppression rate of 70% in Butha-Buthe and Leribe districts, but significantly lower in Mokhotlong district.” There should be a reference cited here with the data source for this statement, and/or some explanation as to why Mokhotlong district is assumed to have a significantly lower rates of viral suppression.

**Response:** *These assumptions were based on our district-wide routine viral load cohort database in Butha-Buthe and Mokhotlong districts.*

*Parts of this routine viral load cohort are published and we added a reference (*[*https://pubmed.ncbi.nlm.nih.gov/34862344/*](https://pubmed.ncbi.nlm.nih.gov/34862344/)*).*

*However, the exact numbers for this age group were extracted specifically by the data manager for the sample size calculation for this study, so they are not published as such.*

12. PDF page 15, line 217. There is a typo of a close bracket ) after the word “Self-reported” which should be deleted

**Response:** *We adapted accordingly.*

13. ”and an ethics committee in Switzerland (Ethikkommission Nordwest- und Zentralschweiz; 2019-00480; June 14, 2019)” can be stated better. It makes this sound like a nondescript, obscure ethics board. Please specify the name of this Swiss committee.

**Response:** *The name of the specific ethics committee is Ethikkommission Nordwest- und Zentralschweiz (see brackets). We modified to clarify.*

14. Please specify the supplement number S1, S2, etc of the CONSORT checklist in the manuscript.

**Response:** *We adapted accordingly.*

RESULTS (including Tables)

1. It goes for all Tables that any category indicated as “Other’ should have a description of what the ‘Other” contains

**Response:** *We adapted accordingly; categories “Other” were removed in current ART regimen and contraceptive used and explained in the footnote for employment.*

2. Table 1 appears quite bulky and can be formatted to make it more palatable to digest.

a. I recommend clustering the employment categories into just 4 or so categories

b. Also consider clustering the marital status into fewer groups eg single, married, then divorced/separated/widowed

c. Cluster the Current ART regimens into NVP-based, EFV-based and LPV/r based. Kaletra is a brand name, please do not use in the manuscript. What is “Other”, and does that include any DTG regimen?

**Response:**

**a./ b.** *We adapted accordingly.*

**c.** *We clustered into NVP / EFV / LPV/r and DTG- based regimens. All 4 “other” regimens were DTG-based.*

3. Table 1-Contraception:

a. For condoms, indicate whether the question specified male or female condoms and provide the disaggregated response

b. I see no indication for IUDs. Is there a space for IUDs? Is that included in Other?

**Response:**

a. *The question included both male and female condoms but as 1 question. So, unfortunately, we are not able to disaggregate the response. We clarified the category.*
b. *IUD was not separately included, however also not found as “other”. Inspection of additional text information of “other” revealed that the only “other” had a DEPO and was therefore added to “injectables”.*
4. “At the 12-month follow-up, 99 of 150 (66%) participants in the intervention versus 95 of 157 (61%) participants in the control arm achieved viral suppression…at what level? <20 or <1,000? Please specify for clarity, here.

**Response:** *We clarified by adding “..below 20 copies/mL”*

5. “At the end of the study, 268/307 (87%) ***participants*** were taking DTG-based ART” ….As opposed to how many, at the beginning? Would be a great piece of info to add here, for immediate comparison.

**Response:** *We added the percentage from table 1:*

At the end of the study, 268/307 (87%) participants were taking DTG-based ART as compared to 13% at the beginning.

DISCUSSION

1. “Three systematic reviews involving studies from 2001 to 2016 evaluated the evidence **on interventions** to improve engagement in HIV care among adolescents and young adults.”

**Response:** *We adapted accordingly.*

2. Suggest edit: “The PEBRA trial offered a similar multicomponent DSD model in **in Lesotho, also in southern Africa; however, it did not**…”

**Response:** *We adapted accordingly.*

3. PDF page 21, Line 336 to 340. The authors state that peer support may be less effective among older participants, but proceed to provide data (effect modifications analysis) on how it may have been more effective in younger participants 15-19 yrs. These are two different things. If the impact (or lack thereof) is described in terms of being less effective among older participants, the data provided must be relevant to older (not younger participants) and the discussion on why would be expected to be mainly from the standpoint of older participants. This just needs a slight edit of the framing of the statement and data provided. Also, cite refs that support your study’s findings that peer support may be less effective for older AYA.

**Response:** *We kindly refer to S4 Table (supplement table 4) that shows lower viral suppression rates in older participants – however, we would like to emphasize again that this was an underpowered treatment effect modification analysis. In our answer to comment/question 3 of Reviewer #3 we give more details about the two age groups. Moreover, this is a hypothesis for the discussion that we cannot prove at this stage, but will further investigate in our secondary paper.*

4. I recommend considering this reference for further support in understanding how peer support works and whether/why some of these insights can explain some of the PEBRA study findings: How Do Psychosocial Interventions for Adolescents and Young People Living With HIV Improve Adherence and Viral Load? A Realist Review <https://scanmail.trustwave.com/?c=22737&d=i-mp41IuQ8uRLI-AwuCBqSq69KHkUpVR_iDmuZSD7A&u=https%3a%2f%2fpubmed%2encbi%2enlm%2enih%2egov%2f35606252%2f>

**Response:** *Thank you for this interesting reference, we added it in the discussion.*

5. “Our pre-planned effect modification analysis supports this hypothesis with a favourable effect **of what?-please specify** on viral suppression…”although these numbers have to be interpreted with caution (Supplementary Table 3).” I suggest outright saying that “although the findings were not statistically significant and thus have to be interpreted with caution.”

**Response:** *We adapted the statement accordingly and worded it more cautiously.*

6. Line 349--*A* literature search…

**Response:** *We adapted accordingly*

7. “However, they proposed a very different intervention than *the* PEBRA model and among a younger population.” How were these interventions “very different”? Explain.

**Response:** *The trial in Uganda proposed a savings-led economic empowerment intervention and the trial in Zimbabwe a community-based caregiver support intervention. We outlined this in the two previous sentences and summarized it again in the sentence in brackets:*

A literature search revealed two other randomized trials that evaluated a support intervention for adolescents living with HIV in sub-Sahara Africa and reported virological outcomes.(ref) One trial, conducted in Uganda(ref), assessed savings-led economic empowerment among 10-16 year old participants, whereas a trial in Zimbabwe (ref) proposed a community-based caregiver support program for children and adolescents aged 6-15 years with a new HIV diagnosis. Both interventions improved viral suppression rates compared to the standard of care. However, they proposed a different intervention (economic and caregiver intervention) than the PEBRA model and among a younger population.

8. “Two major events occurred in Lesotho during the trial period: The large-scale rollout of DTG and the first two waves of COVID-19 **, which triggered social mobility restriction** measures and a brief nation-wide lockdown.” Can you also provide a reference(s) for these events?

**Response:** *We adapted accordingly and added the necessary references*

CONCLUSIONS

9. “ *The* PEBRA trial demonstrates that it is…”

**Response:** *We adapted accordingly.*

10. “As such it supports WHO’s recommendation of incorporating peer-led services not only among adults but also among young people living with HIV.” This is the last part of the manuscript but this is the first time that the WHO recommendations on peer services is mentioned. I suggest citing these recommendations in the Intro; that makes it more relevant to refer to them here, even without citing them in the Conclusions. The WHO reference I am referring to is same as that mentioned in the first comment for the Intro; <https://scanmail.trustwave.com/?c=22737&d=i-mp41IuQ8uRLI-AwuCBqSq69KHkUpVR_nm3s57W6w&u=https%3a%2f%2fwww%2ewho%2eint%2fpublications%2fi%2fitem%2fadolescent-friendly-health-services-for-adolescents-living-with-hiv> *****

**Response:** *We rephrased the conclusion and incorporated the reviewers’ suggestion. The WHO is now cited in the intro but not in the conclusion anymore:*

This trial is one of few randomised clinical trials focusing on differentiated service delivery among young people living with HIV. During the study period of the PEBRA trial viral suppression rates in both groups increased by 9%. Preference-based peer-coordinated care for young people living with HIV, compared to nurse-based care only, did not lead to conclusive evidence for an effect on viral suppression. More large-scale research is needed to understand the effect of peer-led models of care among the youth.
