# Supplementary material for: Effectiveness of a peer educator-coordinated preference-based differentiated service delivery model on viral suppression among young people living with HIV in Lesotho: The PEBRA cluster-randomized trial
Source: PLoS Med. 2023 Jan 3;20(1):e1004150. doi: 10.1371/journal.pmed.1004150 (PMC9810159; doi:10.1371/journal.pmed.1004150)
Supplement: Attachment — Submitted filename: PEBRA_response_to_reviewers_20221111.docx [file pmed.1004150.s007.docx]

**Response to Reviewers**

*Effectiveness of a peer educator-coordinated preference-based differentiated delivery model on viral suppression among young people living with HIV in Lesotho: The PEBRA cluster randomized trial*

*Meanwhile, we also have uploaded our key data onto Zenodo:*

DOI 10.5281/zenodo.7307834

[https://zenodo.org/record/7307834#.Y2wSSXbMI2w](https://zenodo.org/record/7307834#.Y2wSSXbMI2w)

*We have updated our data availability statement accordingly.*

**Requests from the Editor:**

Thank you for your considered and detailed responses to previous editor and reviewer comments.  There are just a few very minor outstanding editorial requests for you to address as detailed below.

***Response:*** *Thank you very much for your positive feedback!*

ABSTRACT

Please include p-values where you have reported 95% Cis

***Response:*** *We adapted accordingly.*

Please include the important dependent variables that are adjusted for in the analyses

***Response:*** *We adapted accordingly:*

The primary endpoint was 12-month viral suppression below 20 copies/mL. Analyses were intention-to-treat and adjusted for cluster, district and sex.

AUTHOR SUMMARY

Thank you for including an author summary which is very well written and very clearly describes your study. Please could you define IT at first use and SMS (also in the main manuscript text) at first use or use alternative terms if you prefer, I leave it you your discretion.

***Response:*** *We adapted accordingly and replaced IT developers everywhere with “mobile application developers”*

INTRODUCTION

In the main manuscript text please substitute the heading “Background” with “Introduction”.  The term background should only be used in the abstract.

***Response:*** *We adapted accordingly.*

REFERENCES

In the introduction of your main manuscript there are some errors regarding in-text reference callouts. In some instances, a space is lacking between the text and the opening parenthesis for example line 110.  In other instances, punctuation precedes the opening parenthesis when it should follow the closing parenthesis, for example line 109.  Please check thoroughly throughout the manuscript and amend where necessary.

***Response:*** *We adapted accordingly.*

**Comments from the academic editor:**

I agree with the decision for minor revisions. One point to clarify - the authors declare no COIs on the first page of the revised manuscript - I want to be sure not to miss author COIs. In light of the above comment please ensure that you have read the journal’s policy regarding competing interests carefully and amend if necessary.

***Response:*** *We confirm that we have read the journal’s policy regarding competing interests and declare no COIs.*
