# Supplementary material for: Effectiveness of a peer educator-coordinated preference-based differentiated service delivery model on viral suppression among young people living with HIV in Lesotho: The PEBRA cluster-randomized trial
Source: PLoS Med. 2023 Jan 3;20(1):e1004150. doi: 10.1371/journal.pmed.1004150 (PMC9810159; doi:10.1371/journal.pmed.1004150)
Supplement: Attachment — Submitted filename: PEBRA_response_to_reviewers_20221114.docx [file pmed.1004150.s008.docx]

**Response to Reviewers**

*Effectiveness of a peer educator-coordinated preference-based differentiated delivery model on viral suppression among young people living with HIV in Lesotho: The PEBRA cluster randomized trial*

**Requests from the Editor:**

1) Thank you for including p-values alongside 95% CIs – please define 95% CI at first use in the abstract (line 62) where you have defined adjusted odds ratio.

Thereafter for clarity suggest reporting the statistical information as follows (aOR 4.12; 95%CI [0.45 to 37.62]; p=0.210).  Please check and revise accordingly throughout the abstract AND main manuscript.

***Response:*** *We adapted accordingly.*

2) Line 87 (author summary) – “…clinics in Lesotho. In the PEBRA model…” suggest making the sentence beginning “In the PEBRA model…” a separate bulleted point.

***Response:*** *We adapted accordingly.*

3) Table 1: the numerical values continued within parentheses appear to be defined in column headers as “n”.  In the table caption these are defined as “(% of those with non-missing data) – which I believe to be the correct definition.  Suggest removing the parentheses where participant numbers are detailed in the row headings and placing below the column title (Total, Control, Intervention) to mitigate against confusion here.  Please also ensure consistent use of “n” in each row.

***Response:*** *We adapted accordingly and hope we understood the comment correctly.*

4) Table 2: the above applies here also.  What do those numerical values within parentheses following “median physical QoL score” depict, for example? All data contained within tables should be easily accessible to the reader.  There are many notes in the table to defer the reader to the caption to try and work things out – perhaps too many?  Please revise this table accordingly to improve accessibility and clarity.

In addition, to help improve transparency of data reporting, where you report adjusted analyses in Table 2 please also include the unadjusted analyses.

***Response:*** *We adapted Table 2, added the explanation to the parentheses following “median physical QoL score” and clarified and reduced the footnotes.*

5) Supplementary tables: please also include unadjusted analyses for data reported in supplementary files, for example Table S2 and S3.  Table S4 - please include include 95% CIs as well as p-values.

***Response:*** *Table S4 is a subgroup analysis and Tables S2 and S3 are sensitivity analyses. We don’t think it makes sense to present unadjusted analyses for these analyses, since the subgroup analysis and the sensitivity analyses should be based on the primary model (adjusted). It would be hard to interpret what these results would mean.*

6) Line 455: Please remove the heading “conclusion”.  The discussion should end with a one paragraph conclusion such that the full discussion reads as one continuous piece of prose, ending in a single paragraph conclusion.

***Response:*** *We adapted accordingly.*
