# Supplementary material for: Effectiveness of a peer educator-coordinated preference-based differentiated service delivery model on viral suppression among young people living with HIV in Lesotho: The PEBRA cluster-randomized trial
Source: PLoS Med. 2023 Jan 3;20(1):e1004150. doi: 10.1371/journal.pmed.1004150 (PMC9810159; doi:10.1371/journal.pmed.1004150)
Supplement: Attachment — Submitted filename: PEBRA_response_to_reviewers_20221124.docx [file pmed.1004150.s009.docx]

**Response to Reviewers**

*Effectiveness of a peer educator-coordinated preference-based differentiated delivery model on viral suppression among young people living with HIV in Lesotho: The PEBRA cluster randomized trial*

**Requests from the Editor:**

1) We understand and accept your rationale for not including unadjusted analyses for the sensitivity and sub-group analyses presented in the supplementary tables.  Thank you for your very clear response which helps to facilitate transparency of data reporting as part of the peer review process.

We did also request the unadjusted analyses for the primary and secondary outcome measures presented in table 2 but did not see them included.  Again, to help us facilitate transparent data reporting, please also include the unadjusted analyses for table 2 ideally in table 2 of the main manuscript (or as a compromise, as a supplementary file).  In the table caption please indicate which factors are adjusted for in the analyses.

***Response:*** *Thank you for clarifying. We don’t think it is wise to remove the clustering and stratification variables (clinic & district) since these are design variables, representing the clustering of the data in a multilevel model, but we agree that we should have provided unadjusted analyses for sex. We adapted Table 2 accordingly. Thank you for your thorough review.*
